# Virtual zero-photon catalysis for improving continuous-variable quantum key distribution via Gaussian post-selection

**DOI:** 10.1038/s41598-020-73379-4

**Published:** 2020-10-16

**Authors:** Hai Zhong, Ying Guo, Yun Mao, Wei Ye, Duan Huang

**Affiliations:** 1 School of Computer Science and Engineering, Central South University, Changsha, 410083 China; 2 State Key Laboratory of Advanced Optical Communication Systems and Networks, Shanghai Jiao Tong University, Shanghai, 200240 China

**Keywords:** Quantum information, Quantum optics

## Abstract

Quantum catalysis is a feasible approach to increase the performance of continuous-variable quantum key distribution (CVQKD), involving the special zero-photon catalysis (ZPC) operation. However, in the practical point of view, the improvement effect of this operation will be limited by the imperfection of the photon detector. In this paper, we show that the ZPC operation at the sender can be simulated by a post-selection method without implementing it in practical devices. While performing this virtual version of ZPC in CVQKD, we can not only reach the ideal case of its practical implementation with minimal hardware requirement, but also keep the benefit of Gaussian security proofs. Based on Gaussian modulated coherent state protocols with achievable parameters, we enhance the security of the proposed scheme from the asymptotical case to the finite-size scenario and composable framework. Simulation results show that similar to the asymptotical case, both the maximal transmission distance and the tolerable excess noise of virtual ZPC-involved CVQKD outperform the original scheme and the scheme using virtual photon subtraction while considering finite-size effect and composable security. In addition, the virtual ZPC-involved CVQKD can tolerate a higher imperfection of the detector, enabling its practical implementation of the CVQKD system with state-of-the-art technology.

## Introduction

Quantum key distribution (QKD)^1,2^, the best known application of quantum communication, enables two remote legal parties, called Alice and Bob, to establish secret keys over an insecure quantum channel controlled by an eavesdropper (Eve). One implementation approach of QKD is to encode the information in the state of single photon^3^, such as polarization. However, this discrete-variable (DV) QKD requires single-photon detector in practice, which may bring about challenges for the commercial popularization of DVQKD because of its cost and limited detection efficiency. Alternatively, continuous variable (CV) QKD, which encodes the information in the quadratures ($\hat{x}$ and $\hat{p}$)^4–7^, can be implemented with coherent state and thus be compatible with standard telecommunication optical networks. In theory, its unconditioned security has been proven to be secure against general collective attacks^8,9^. In practice, high secret key rate and long transmission distance of CVQKD have also been achieved^10–16^. Nevertheless, restricted by the limited data post-processing speed and the sensitivity to excess noise, the transmission distance of CVQKD can not catch up with its DV counterpart^17^.

One of feasible methods that can improve the transmission distance of CVQKD is the use of linear amplifiers, including deterministic and probabilistic amplifiers. Deterministic linear amplifier^18–20^, such as phase-insensitive amplifier, can amplify each incoming signal deterministically so that the performance of CVQKD can be improved^21–23^. However, actual deterministic linear amplifier not only amplifies the incoming signal and its own noise linearly, but also unavoidably adds extra noise on the signal, which reduces the signal-to-noise ratio. Alternatively, probabilistic amplifier, such as noiseless linear amplifier, amplifies the incoming signal linearly with a certain probability, which is heralded, but introduces less even no noise^24–28^. Such a noiseless linear amplifier is demonstrated to extend the transmission distance of CVQKD^29,30^. However, a successfully heralded event of noiseless amplifying needs a single-photon event on photon detector, which renders its practical employment challenging. Interestingly, such probabilistic amplifying can be emulated by post-selection method, namely virtual noiseless amplification^31^. Although this virtual method makes possible its application for CVQKD, the post-selection probability function of it is diverging, which will complicate the security analysis^32^. Recently, the combination of deterministic and noiseless linear amplifier has been investigated theoretically and experimentally^33,34^. Such heralded linear amplifier can also be applied to improve the performance of CVQKD^35^.

Another viable approach that can improve the transmission distance of CVQKD is the employing of quantum operation, including photon subtraction, quantum scissors and quantum catalysis (QC) operation. The photon-subtraction and quantum scissors operations are non-Gaussain operation. They degrade the secret key rate in short distance but increase the maximal transmission distance of CVQKD^36–43^. Yet, there exists a possible drawback when taking photon number resolving detector (PNRD) into account. Fortunately, the propose of virtual photon-subtraction for CVQKD enables the photon-subtraction operation to a worthy of practical implementation^44–47^. However, rigorous security proof of such non-Gaussian protocol is still an open problem. Attractively, the quantum photon catalysis operation, especially for zero-photon catalysis (ZPC), compared with the photon-subtraction operation applied to CVQKD, has larger success probability as well as better performance improvement in terms of secret key rate, transmission distance and tolerable excess noise^48–50^. Nevertheless, this approach also needs a PNRD or an on/off detector to herald a successful quantum photon catalysis operation, which degrades its practical significance.

To remove the above-mentioned imperfections of the photon detector, in this paper, we propose a scheme of virtual zero-photon catalysis (VZPC)-based CVQKD, which can be implemented via Gaussian post-selection at the sender. One advantage of this method of VZPC is that it can not only emulate an ideal ZPC operation but also implement it without practical devices. Another advantage is that the post-selection probability function or the acceptance function is Gaussian and bounded, and thus our proposed scheme remains equivalent to an effective deterministic Gaussian protocol, which can follow all the Gaussian security proofs as well as the corresponding conventionally Gaussian data post-processing. Furthermore, considering the imperfection of the detector, we expand the security of the proposed scheme from the asymptotical case to the finite-size scenario and composable framework based on Gaussian modulated coherent state protocol and experimentally reachable parameters, in homodyne and heterodyne detection configurations. The VZPC-based CVQKD not only outperforms the original scheme and the scheme with virtual photon subtraction, but also has a higher toleration of the imperfection of the detector.

## Results

### The VZPC-based CVQKD

Quantum catalysis can be applied to facilitate the conversion of the target ensemble^51^ and enhance the coherence of the coherent state^52^, which could prevent the loss of information effectively. It has been demonstrated theoretically that the performance of CVQKD can be improved when acting quantum catalysis operation on it, in both the Einstein-Podolsky-Rosen (EPR)-based scheme^48^ and the locally generated local oscillator (LLO)-based scheme^49^. To make our description self-contained, in this section, we introduce the characteristics of quantum catalysis operation, and then propose the VZPC-based CVQKD.

**Figure 1:**
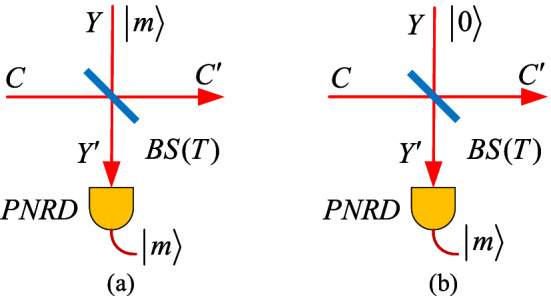
Illustrations of the quantum operation. **(a)** Quantum catalysis operation. **(b)** Photon subtraction operation. BS, beam splitter. PNRD, photon number resolving detector.

The characteristics of quantum catalysis operation can be illustrated in Fig. 1a. An auxiliary mode *Y* with number state $|m\rangle$ interacts with the input mode *C* at the beam splitter (BS) with a transmittance *T*. The output $Y'$ of mode *Y* then launches into an ideal PNRD to herald whether a successful quantum catalysis operation happened or not. If there remains *m* photons registered in the PNRD, we call the process above a successful *m*-photon quantum catalysis operation, which is represented by an equivalent operator $\hat{C}_m$^48^,

1 $$\begin{aligned} \hat{C}_m= & {} \frac{\partial ^m}{m!\partial \chi ^m}\left\{ \frac{T^m}{1 - \chi }\left( \frac{\sqrt{T} - \chi /\sqrt{T}}{1 - \chi }\right) ^{c^\dag c}\right\} _{\chi = 0}, \end{aligned}$$

where *T* is the transmittance of the BS in Fig. 1a, $c^\dag$ and *c* are the creation and annihilation operators of the input state in mode *C*. Note that, for the auxiliary modes *Y* and $Y'$ of quantum catalysis operation, the input and output photon number seems like invariable, which means the detection result of the *PNRD* must be equal to the photon number of mode *Y* and is different from the *m*-photon subtraction operation (*m* > 0) shown in Fig. 1b. The post-selection state of $C'$ for ZPC is conditioned on the 0 photon event on the *PNRD* while for *m*-photon subtraction is conditioned on the *m* (*m* > 0) photon event on the *PNRD*.

**Figure 2:**
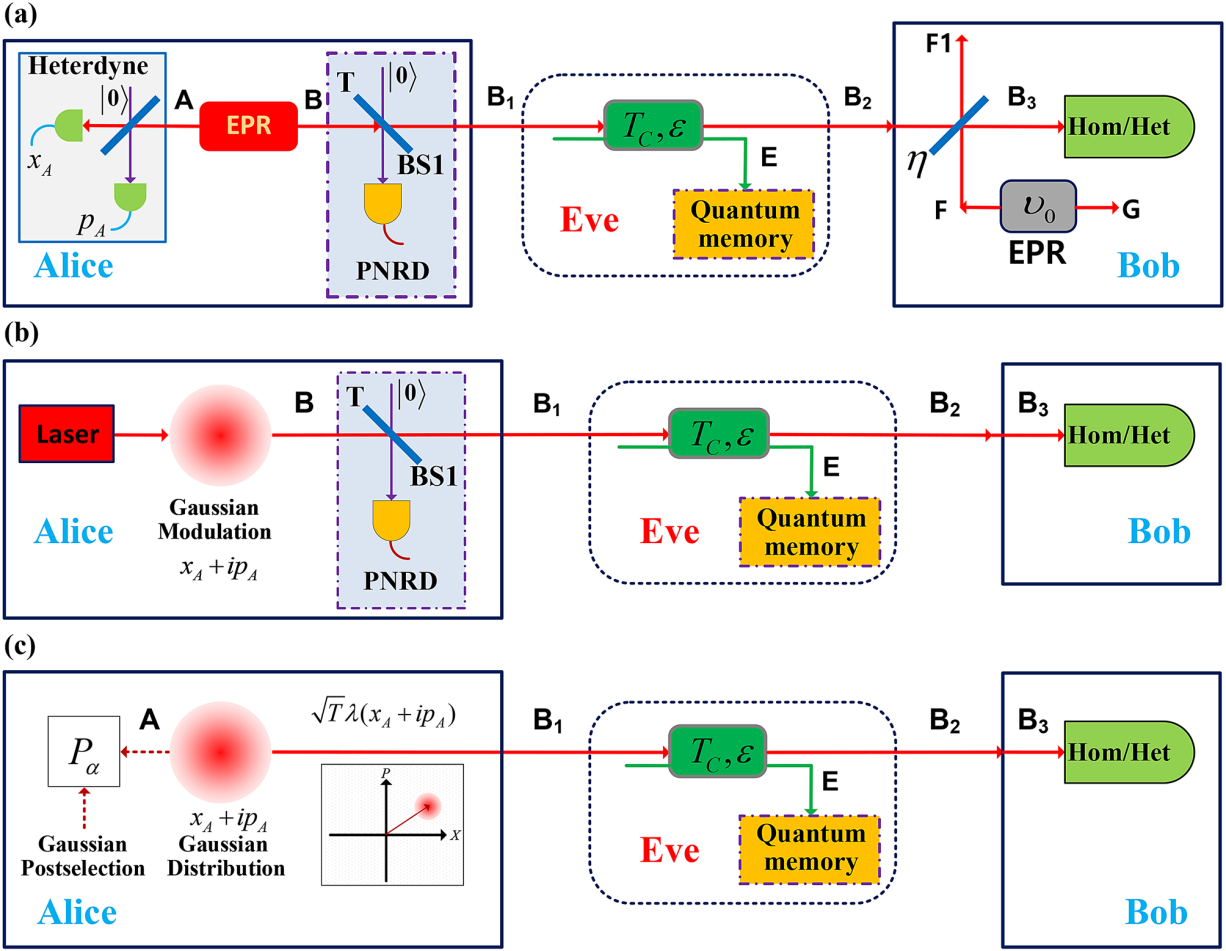
**(a)** Equivalent entanglement-based (EB) scheme of CVQKD with the ZPC. **(b)** Prepare-and-measure (PM) scheme of CVQKD with the ZPC. **(c)** PM scheme of CVQKD with the VZPC. $\eta$, the quantum efficiency if the detector. *T*, the transmittance of the BS1. $P_{\alpha }$, acceptance probability. $\upsilon _0$, the equivalent variance of EPR state modelling the electronic noise of the detector. $\upsilon _0 = \eta \chi _{det}/(1 - \eta )$ for homodyne detector and $\upsilon _0 = (\eta \chi _{det} - 1 )/(1 - \eta )$ for heterodyne detector.

The quantum catalysis operation has larger success probability than the photon subtraction operation, and hence the ZPC-based CVQKD has better performance improvement in terms of secret key rate, transmission distance and tolerable excess noise^48,49^, especially for the ZPC. Therefore, in this work, we mainly focus on the ZPC operation. The specific application scheme of the ZPC in the equivalent entanglement-based (EB) CVQKD and its corresponding prepare-and-measure (PM) scheme can be simply described in Fig. 2a, b, respectively. However, from a practical point of view, the requirement of a PNRD or an on-off detector brings about practical difficulties. The imperfection of the detector and the augment to CVQKD system’s complexity reduce the attractiveness of quantum catalysis operation. To overcome the difficulties mentioned above, in what follows, we suggest an equivalent post-selection method to emulate the ZPC operation in CVQKD with Gaussian modulated coherent state protocol. By using this method, one can not only enjoy the perfect ZPC operation but also remove the problem of increasing the system’s complexity.

Now, let us begin the description of the equivalent post-selection method with the EB scheme of the ZPC-based CVQKD. As shown in Fig. 2a, Alice prepares a pair of EPR state, including two modes *A* and *B* with a modulation variance *V*, i.e.

2 $$\begin{aligned} |\Phi \rangle _{AB} = \sqrt{1 - \lambda ^2}\sum _{n = 0}^{\infty }\lambda ^n|n,n\rangle _{AB}, \end{aligned}$$

where $\lambda = \sqrt{(V - 1)/(V + 1)}$. She retains mode *A* of the two modes and sends the other mode *B* to Bob. For the preserved mode *A*, Alice performs heterodyne detection on it and gets the results {$x_A$, $p_A$}, which obeys Gaussian distribution with probability distribution $P_A = \frac{1}{2\pi (V + 1)}e^{-(x_A^2 + p_A^2)/2(V + 1)}$^53^. It is known that heterodyne detection on mode *A* will project mode *B* onto a coherent state $|\alpha \rangle$ with $\alpha = x_b + ip_b = \lambda (x_A - ip_A)$^54^. Consequently, the mixed state $\rho _{B}$ can be given by

3 $$\begin{aligned} \rho _B = \int \mathbf {d}x_b\mathbf {d}p_bP_{b}|\alpha \rangle \langle \alpha | = \int \mathbf {d}x_A\mathbf {d}p_AP_{A}|\alpha \rangle \langle \alpha |, \end{aligned}$$

where $P_b = \frac{1}{2\pi (V - 1)}e^{-(x_b^2 + p_b^2)/2(V - 1)}$^53^. Before Alice sends mode *B* to Bob, she performs the ZPC operation on mode *B* and yields the mode $B_1$ if this operation is successful. Therefore, after the BS1, the mixed state of mode $B_1$ undergone a successful ZPC operation can be given by

4 $$\begin{aligned} \rho _{B_1} = \frac{1}{P_Z}\int \mathbf {d}x_b\mathbf {d}p_be^{|\alpha |^2(g^2 - 1)}P_b|g\alpha \rangle \langle g\alpha | = \int \mathbf {d}x_A\mathbf {d}p_AP_A'|g\alpha \rangle \langle g\alpha | \end{aligned}$$

with

5 $$\begin{aligned} P_A' = e^{|\alpha |^2(g^2 - 1)}P_A/P_Z = \frac{1}{2\pi P_Z(V + 1)}e^{-\frac{x_A^2 + p_A^2}{2P_Z(V + 1)}}, \end{aligned}$$

where $P_Z$ is the success probability of the ZPC operation. It can be obtained by the relationship of $\mathrm {Tr}(\rho _{B1})=1$, given by $P_Z = \frac{2}{1 - VT + V + T}.$ Comparing with the state $\rho _{B_1}$ in Eq. (4) and the state $\rho _B$ in Eq. (3), one can find two differences between them. One is the additional weighting function $W = e^{|\alpha |^2(g^2 - 1)}/P_Z$, which leads to an acceptance probability $P_{\alpha } = e^{|\alpha |^2(g^2 - 1)}$ for each pair of measured results $\{x_A,p_A\}$. The total acceptance probability for all the data measured by Alice is $\int \mathbf {d}x_A\mathbf {d}p_AP_{\alpha }P_A = P_Z$, resulting in the success probability of the ZPC operation. The other one is the rescaling of the mean value of the coherent state from $\alpha$ to $g\alpha$ ($\sqrt{T}\alpha$). Although the ZPC operation is probabilistic, $P_{\alpha }$ is Gaussian so that $P_A'$ is still a Gaussian distribution, and thus this filter is Gaussian in the sense that it converts one Gaussian state into another Gaussian state, which means the ZPC operation preserves Gaussianity while the *m*-photon quantum catalysis operation ($m >0$) is non-Gaussian^55^. As the heterodyne detection and the ZPC operation are conducted on two different modes *A* and *B*, these two processes can be commuted with each other. Thus, we get an equivalent VZPC through Gaussian post-selection of Alice’s each measurement result with an acceptance probability $P_{\alpha }$ after exchanging the heterodyne detection and the ZPC operation. To be more specific, the PM version of the VZPC-based CVQKD, as shown in Fig. 2c, can be summarized as follows:

Step 1: Alice prepares a coherent state $|\alpha \rangle$ with $\alpha =\sqrt{T}\lambda (x_A + p_A)$ and sends it to Bob, where $x_A$ and $p_A$ obey Gaussian distribution with zero mean and variance of $(V + 1)$.
Step 2: Bob receives the coherent state sent by Alice and performs homodyne or heterodyne detections. The measurement results are $x_B$ and $p_B$, respectively.
Step 3: After performing many rounds of step 1 and 2, Alice and Bob preserve enough data. Alice uses the acceptance probability to determine which data will be accepted and reveals them to Bob.
Step 4: Using the accepted data determined by step 3, Alice and Bob accomplish the conventional post-processing steps, involving parameter estimation, information reconciliation and privacy amplification.

Since the Gaussian post-selection can not change the Gaussianity of the data that Alice finally determined for generating secret keys, the suggested scheme remains equivalent to an effective deterministic Gaussian protocol, which can follow all the Gaussian security proofs as well as the corresponding conventionally Gaussian data post-processing. Note that since $0 < g \le 1$, the acceptance probability function $P_{\alpha }$ is bounded. As a result, for arbitrary probability distribution $P(\alpha )$ of $\alpha$, the integration $\int \mathbf {d} \alpha ^2 P_{\alpha }P(\alpha )$ is convergent. Therefore, differing from the virtual noiseless amplification^31^, there is no divergence problem for the VZPC-based CVQKD.

### Performance analysis

In this section, based on Gaussian modulation of the quadratures of coherent states, we show the performance of our scheme for the security frameworks of asymptotic, finite-size and composable scenarios under a collective Gaussian attack assumption.

In order to derive the analytical expression of the secret key rate, we need get the covariance matrix $\Gamma _{AB_3}$ of the state $\rho _{AB_3}$ in the equivalent EB-based version of our scheme. The covariance matrix of the EPR state $\rho _{AB}$ that Alice prepared can be given by^56^

6 $$\begin{aligned} \Gamma _{AB} = \left( \begin{array}{cc} V\mathbf {I} &{} \sqrt{V^2 - 1}\sigma _Z\\ \sqrt{V^2 - 1}\sigma _Z &{} V\mathbf {I} \end{array} \right) , \end{aligned}$$

where $$\mathbf {I} = \bigg (\begin{array}{cc} 1 &{} 0 \\ 0 &{} 1 \end{array}\bigg )$$ and $$\sigma _Z = \bigg (\begin{array}{cc} 1 &{} 0 \\ 0 &{} -1 \end{array}\bigg )$$. After performing the ZPC operation on mode *B*, the covariance matrix of the yield state $\rho _{AB_1}$ can be expressed as

7 $$\begin{aligned} \Gamma _{AB_1} = \left( \begin{array}{cc} V_A\mathbf {I} &{} C_1\sigma _Z\\ C_1\sigma _Z &{} V_{B_1}\mathbf {I} \end{array} \right) , \end{aligned}$$

where

8 $$\begin{aligned} V_A= & {} V_{B_1} = V_a + 1 = \frac{1 + VT + V - T}{1 - VT + V + T}, V_a = \frac{2T(V - 1)}{1 - VT + V + T}. \end{aligned}$$

9 $$\begin{aligned} C_1= & {} \frac{2\sqrt{T(V^2 - 1)}}{1 - VT + V + T}, \end{aligned}$$

The explicit derivation of the above expressions and the covariance matrix $\Gamma _{AB_3}$, containing the cases of asymptotic, finite-size and composable scenario, are shown in Supplementary S1. With the covariance matrices obtained in Supplementary S1, we can calculate the secret key rates using techniques shown in Refs.^8,9,48,56^ for asymptotic, finite-size and composable security, as shown in Supplementary S2. For comparison, in what follows, we also show the simulation results of CVQKD with virtual photon subtraction (VPS). Its computing of covariance matrixes and secret key rates under asymptotic, finite-size and composable security are shown in Supplementary S3. Since the CVQKD with virtual 1-photon subtraction (V1-PS) has the optimal performance improvement^44^, we only show results of V1-PS-based CVQKD here. Note, as the 1-PS operation is non-Gaussian, all the calculated results for it are lower bounds based on the Gaussian hypothesis according to the extemality of Gaussian quantum states^57^.

**Figure 3:**
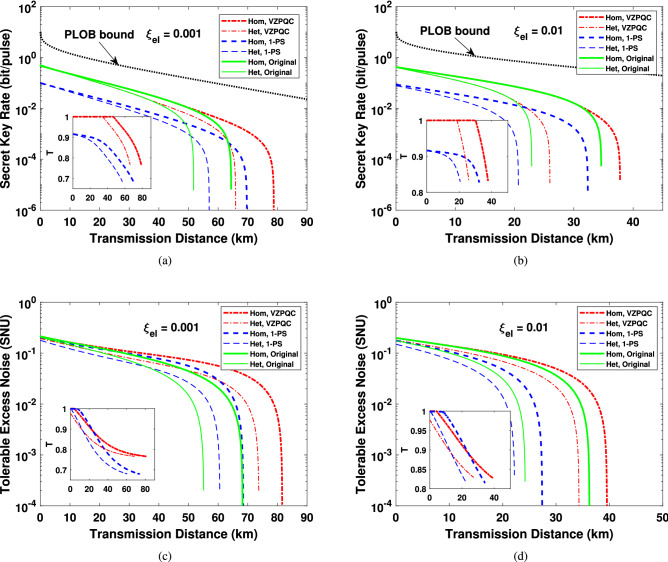
The simulation results under asymptotical security. **(a)**, **(b)** The results of secret key rate for the VZPC-based scheme, the 1-photon-subtraction-based scheme and the original scheme, when the electronic noise of the detector are 0.001 and 0.01, respectively. The optimal value of *T* at each distance are shown in the insets. **(c)**, **(d)** The tolerable excess noise and the corresponding values of *T* (the insets) for the proposed scheme and the original scheme, when $\xi _{el}$ is equal to 0.001 and 0.01, respectively. The black dotted line represents the PLOB bound. 1-PS: 1-photon subtraction.

#### Asymptotic security

We now give the asymptotical security of the VZPC-based CVQKD. The global parameters that are used for simulation are the variance *V* of the equivalent EPR state, the transmission efficiency $T_C$ and the excess noise $\xi$ of the quantum channel, the quantum efficiency $\eta$ and electronic noise $\xi _{el}$ of the detector, the reconciliation efficiency $\beta$ of the reverse reconciliation phase. The parameters $\xi$, $\eta$ and $\beta$ are fixed to values $\xi = 0.01$ (in shot noise units), $\eta = 0.6134$, $\beta = 0.96$^14^. The variance *V* takes the value 20 (in shot noise units), while we set the electronic noise of the detector $\xi _{el}$ to 0.01 or 0.001 (in shot noise units)^11^. In addition, the channel transmission efficiency is written as $T_C = 10^{-\alpha L/10}$, where $\alpha = 0.2$ dB/km is the loss coefficient of optical fibers, and *L* is the fiber length.

**Figure 4:**
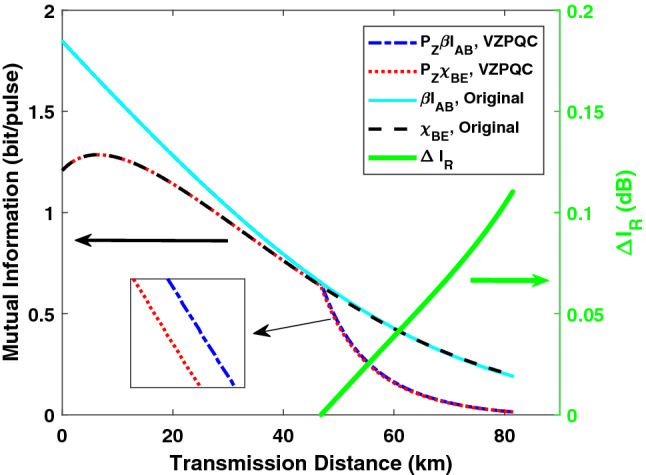
The final mutual information $P_Z\beta I_{AB}$ between Alice and Bob and $P_Z\chi _{BE}$ between Bob and Eve, and the result of $\Delta I_{R}$ as a function of transmission distance (green solid line). Here, we only show the results of homodyne detection when $\xi _{el} = 0.001$.

The simulation results for homodyne and heterodyne detection are shown in Fig. 3a, b, where dash-dotted, dashed and solid lines are related to the results of the VZPC-based CVQKD, the V1-PS-based CVQKD and the original CVQKD, respectively. The dotted lines are the fundamental benchmark of CVQKD, i.e. PLOB bound^58^. Note that $T = 1$ represents catalysis operation is not performed. From the above results we can find that similar to the results in Ref.^48^, our proposed scheme can improve the maximal transmission distance and outperforms the V1-PS-based CVQKD CVQKD, while considering the imperfection of the detector. When the electronic noise arises to 0.01, the V1-PS-based CVQKD can no longer improve the transmission distance, whereas our proposed scheme is still effective. Actually, the ZPQC operation will decrease not only the final mutual information $P_Z\beta I_{AB}$ shared by Alice and Bob but also the stolen information $P_Z\chi _{BE}$ available to Eve on Bob’s key, as shown in Fig. 4. Interestingly, compared to the former, the latter has a relatively larger degradation, which enables the secret key rate to remain positive in the long-distance regime, as shown in Fig. 4, where the vertical axis represents the ratio between the decrement $\Delta \chi _{BE}$ of $P_Z\chi _{BE}$ and the decrement $\Delta I_{AB}$ of $P_Z\beta I_{AB}$ (in the unit of dB), which is defined as $\Delta I_{R} = 10\mathrm {log}_{10}(\Delta \chi _{BE}/\Delta I_{AB})$. Furthermore, from the green solid line in Fig. 4, we can find that $\Delta I_{R}$ increases with the transmission distance, which enables our proposed system to reach a longer maximal transmission distance.

In addition, the electronic noise of the detector at the receiver has a significant effect on our suggested scheme. Larger electronic noise not only degrades the entire performance significantly, but also decreases the performance improvement of CVQKD with the VZPC, which shows the possibly large limit of detector to practical system. Besides, except within very short transmission distance (smaller than 10 km), the performance of homodyne detection is better than heterodyne detection for $\xi _{el} = 0.001$. While the electronic noise of detector arising to 0.01, the performance of homodyne detection is always better than heterodyne detection. The reason is a extra unit of shot noise introduced by the heterodyne detection.

We also give the tolerable excess noise for the given parameters above, as shown in Fig. 3c, d, where the insets mean the corresponding optimal value of *T*. The tolerable excess noise is larger than the original scheme and the V1-PS-based scheme. Similar the secret key rate results in Fig. 3b, for the electronic noise up to 0.01, the tolerable noise can no longer be improved for the V1-PS-based protocol. These mean a more robust CVQKD system can be obtained with the VZPC, by optimizing the parameter *T*.

#### Finite-size analysis

**Figure 5:**
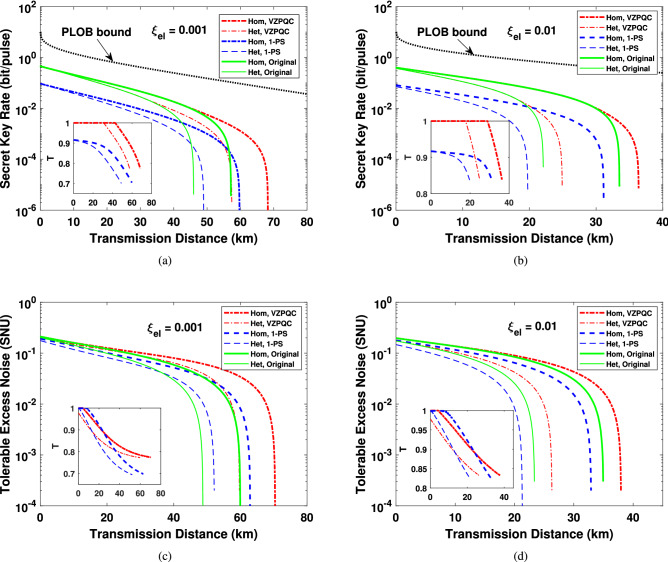
The simulation results considering finite-size effect. **(a)**, **(b)** The results of secret key rate for the VZPC-based scheme, the 1-photon-subtraction-based scheme and the original scheme, when the electronic noise of the detector are 0.001 and 0.01, respectively. The rate *r* is optimized and the optimal value of *T* at each distance are shown in the insets. **(c) **, **(d)** The tolerable excess noise and the corresponding values of *T* (the insets) for the proposed scheme and the original scheme with a fixed $r = 5/7$, when $\xi _{el}$ is equal to 0.001 and 0.01, respectively. The black dotted line represents the PLOB bound.

In the practical implementation of CVQKD, the amount of signal uses is not infinite, which means that we must consider the finite-size effect of the proposed scheme. Moreover, some of the signal uses are discarded in the post-selection process, which will decrease the final signals used for parameter estimation and secret key generation. This post-selection process may generate significant impact on finite-size regime. Therefore, for protocols using post-selection, finite-size analysis is of great significance for practical application. In the following, we expand our suggested VZPC-based CVQKD to the finite-size case, which is more in line with the practical situation. We note that the final simulation results are similar to that of the asymptotic case, as shown in Fig. 5, where the block size *N* is set to be $10^9$, the parameters $\epsilon _{EP}$, $\epsilon _{PA}$ and $\epsilon _{sm}$ are all set to be $10^{-10}$^8^. The rate $r = m/N$, which is the rate of signals uses used for parameter estimation, is optimized for each distance in Fig. 5a, b, while it is fixed with $r=5/7$^11^ for Fig. 5c, d. It is visible that not only the maximal transmission distance but also the tolerable excess noise can be improved for the VZPC-based CVQKD when considering the finite-size effect, although the degree of improvement is slightly decreased. Moreover, our proposed scheme outperforms the V1-PS-based scheme as well. While the block size *N* is up to $10^{12}$, the results considering finite-size effect will closely approach that of the asymptotical case.

**Figure 6:**
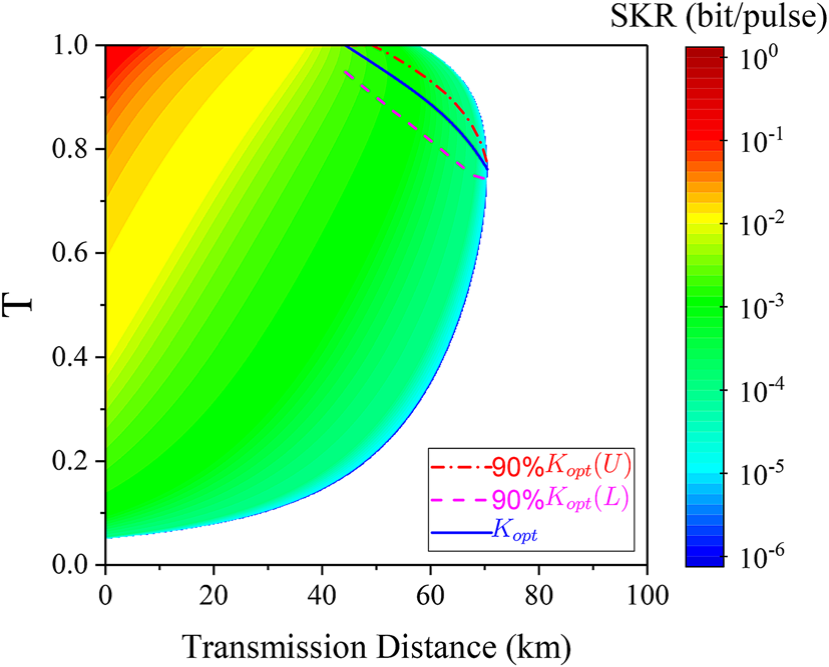
The secret key rate as a function of transmission distance and *T*. The blue solid line denotes the optimal value of *T* to acquire the optimum secret key rate ($K_{opt}$). The red dash-dotted line represents the upper bound of *T* at each distance while the secret key rate remains up to 90% of its optimum. The magenta dashed line denotes the lower bound of *T* at each distance while the secret key rate remains up to 90% of its optimum. The variance *V* and the electronic noise are set to 20 and 0.001. The block size $N = 10^9$ and $r = 5/7$.

From a practical point of view, the evaluation of the optimal value of *T* will be under certain region. If the fluctuation of the estimated value of *T* has large impact on the proposed CVQKD system with the VZPC, complicated implementations will be required to acquire an accurate evaluation of *T*, leading to the employment of the VZPC non of worthy. Therefore, we inspect the variation of secret key rate under finite-size scenario when the parameter *T* diverging from its optimal value a certain range. Fortunately, the secret key rate changes slowly with the fluctuation of *T* at each distance around its optimum value $K_{opt}$, as illustrated in Fig. 6, where $V = 20$ and $\xi _{el} = 0.001$. We can get that higher than 90% of the optimum secret key rate ($K_{opt}$) can be obtained while *T* changing in a relative large region, which is within the scope between the dashed line ($K_{opt}(L)$) and the dash-dotted line ($K_{opt}(U)$). Similar results can be acquired for the case of asymptotical and composable security and we do not show it for simplicity.

**Figure 7:**
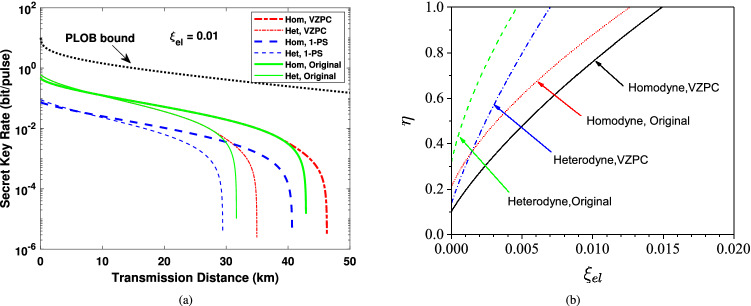
**(a)** The secret key rate as a function of transmission distance considering finite-size effect. The detector’s quantum efficiency $\eta$ is equal to 1. **(b)** To obtain a secret key rate of $10^{-4}$ bit/pulse, the detector’s quantum efficiency as a function of electronic noise under the finite-size security. Here we set the transmission distance $L = 40$ km. The block size $N = 10^9$ and $r = 5/7$ for all these two figures.

Finally, we focus on the comparison of the extent of the effect on our proposed CVQKD scheme between the detector’s quantum efficiency and electronic noise. We depict the secret key rate simulation results in Fig 7a with electronic noise $\xi _{el} = 0.01$, while the quantum efficiency the detector is equal to 1. We find that even the quantum efficiency is up to the perfect case, the performance remains a big gap compared to the case of $\xi _{el} = 0.001$ and $\eta = 0.6134$ (see Fig. 5a), which reflects that the detector’s electronic noise is a more sensitive parameter than its quantum efficiency, either homodyne or heterodyne detection. To make the results more clear, we draw the secret key rate as a function of detector’s quantum efficiency and electronic noise in Fig. 7b, where the transmission distance is 40 km. Since there is little even no performance improvement for 1-photon subtraction operation, we don’t show its results here. We find that the imperfection toleration of homodyne detection is better than that of the heterodyne detection. In particular, compared with the original CVQKD protocol, our suggested scheme can tolerate a higher imperfection of the detector, especially for homodyne detection. These results related to the detector are also applied to the case under previous asymptotical security and the latter composable security.

#### Composable security

The composable security is of great significance for CVQKD to apply in more complex and unpredictable environments, whereas the finite-size analysis is a foundation for the practical implementation of CVQKD. Actually, for realistical application of CVQKD in complex environments, there exists a probability that the finally generated secret keys do not meet the requirement of the CVQKD protocol, i.e. the protocol has “flaw”. The composable security can relax the problem of how to evaluate the degree of this “flow” of a CVQKD protocol, and can also solve the problem of evaluating “flow degree” while the flowed keys are applied in other subsequent protocols. Therefore, it is of importance that whether the proposed scheme can also be applied to real complex environments and composed with other composable secure protocols. Composable security against collective attack for CVQKD has been proven in Ref.^9^, which further implies composable security against general security combining the de Finetti theorem or postselection technique. Here, we give a further analysis of the proposed scheme in composable security framework against collective attacks, exploring the feasibility of the scheme under general security framework and therefore demonstrating the practical significance of it. All the values of the security parameters are chosen as follows^9^:

10 $$\begin{aligned} \epsilon _{sm} = \bar{\epsilon } = 10^{-21}, \epsilon _{PE} = \epsilon _{cor} = \epsilon _{ent} = 10^{-41}. \end{aligned}$$

**Figure 8:**
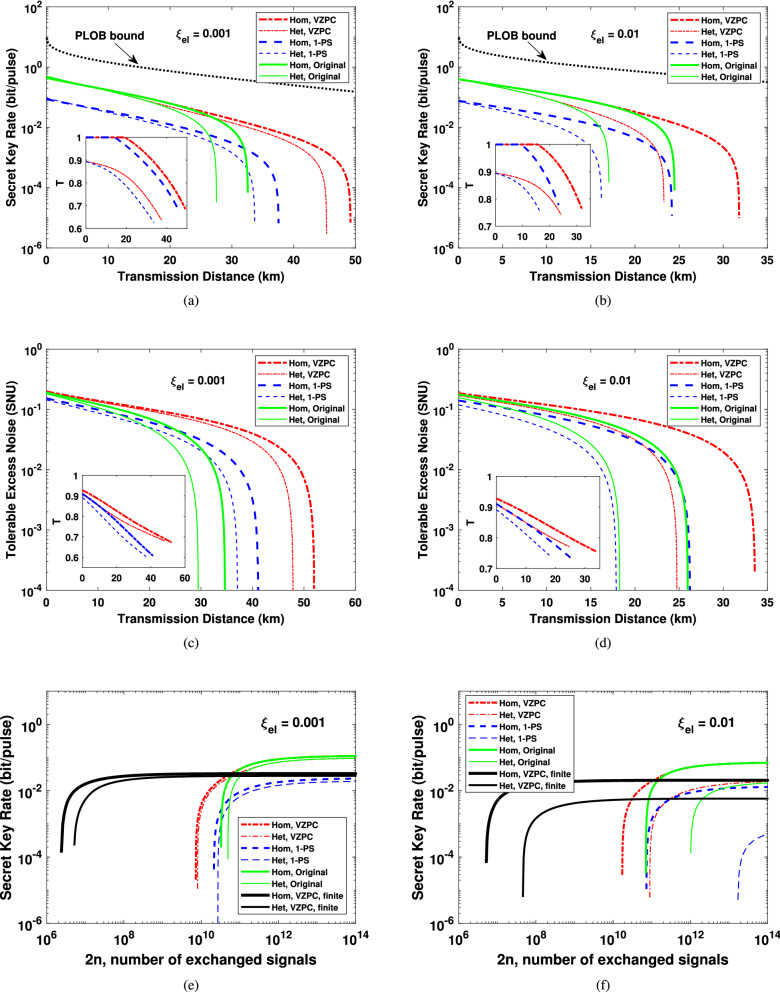
The simulation results considering composable security. **(a)**, **(b)** The results of secret key rate for the VZPC-based scheme, the 1-photon-subtraction-based scheme and the original scheme, when the electronic noise of the detector are 0.001 and 0.01, respectively. The optimal value of *T* at each distance are shown in the insets. **(c)**, **(d)** The tolerable excess noise and the corresponding values of *T* (the insets) for the proposed scheme and the original scheme, when $\xi _{el}$ is equal to 0.001 and 0.01, respectively. **(e)**, **(f)**Expected secret key rate as a function of 2*n* for 20 km transmission distance, when $\xi _{el}$ is equal to 0.001 and 0.01, respectively. The black dotted line represents the PLOB bound.

We plot the numerical results of secret key rate and tolerable noise as well as the corresponding optimal value of *T* in Fig. 8a–d, where the total exchanged signals between Alice and Bob is set to be $2n = 10^{11}$. From the simulation results, we can see that similar to the finite-size case, the maximum transmission distance can be improved in composable security framework as well. However, although the block size is up to $10^{11}$, the maximally reachable transmission distance is even less than that of the finite-size case with $N = 10^{9}$. The performance of finite-size case will approach to the asymptotical case while the block size reaches $10^{11}$, whereas the block size should come up to $10^{14}$ for composable security. Moreover, the results will be even worse for composable security with a lower block size. If the block size $2n = 10^{10}$ and $\xi _{el} = 0.001$ (not shown), only less than 24 km and 22 km can be reached for our proposed scheme with homodyne and heterodyne detection, respectively. For $2n = 10^{10}$ and $\xi _{el} = 0.01$, the results are even worse, merely less than 17 km and 12 km can be reached for our proposed scheme while performing homodyne and heterodyne detection. Non positive key rate can be obtained for all cases when $2n = 10^9$. Therefore, it is more sensitive to the block size for the practical system under composable security framework. We plot the secret key as a function of 2*n*, the number of exchanged signals, in Fig. 8e, f, where the transmission distance is 20 km. From these results we can conclude that compared to the V1-PS-based and the original CVQKD protocol, our proposed scheme can tolerate smaller limit of block size. Meanwhile, in Fig.  8e, f, we also show the corresponding results of our proposed scheme under finite-size security (two black solid lines), with a fixed rate $r = 5/7$. Obviously, compared with the finite-size case, composable security has a higher security level and thus it requires much larger number of channel uses.

## Discussion

We have proposed an approach, virtual zero-photon catalysis, to emulate the ideal zero-photon catalysis operation without practical devices in coherent-state CVQKD, which can be realized by Gaussian post-selection according to Alice’s data. The post-selection probability function or the acceptance function is Gaussian and bounded, which guarantees our proposed protocol remains equivalent to an effective deterministic Gaussian protocol, and thus enables it to follow all the Gaussian security proofs as well as the corresponding conventionally Gaussian data post-processing. The simulation results show that by optimizing the main parameter, i.e., the transmittance *T* of Alice’s BS1, both the maximal transmission distance and the tolerable excess noise can be increased and outperform the V1-PS-based CVQKD scheme, which is true for the asymptotical security and the security considering finite-size effect. By comparing the results of homodyne and heterodyne detection, additionally, we find that the performance of homodyne detection is superior to that of the heterodyne detection. Attractively, the CVQKD using virtual zero-photon quantum catalysis can tolerate a higher imperfection of the detector, especially for homodyne detection. These results indicate that our proposed scheme offers an opportunity of practically implementing the zero-photon catalysis on CVQKD systems.

## Supplementary information

Supplementary Information.
