# Supplementary material for: Virtual zero-photon catalysis for improving continuous-variable quantum key distribution via Gaussian post-selection
Source: Sci Rep. 2020 Oct 16;10:17526. doi: 10.1038/s41598-020-73379-4 (PMC7567868; doi:10.1038/s41598-020-73379-4)
Supplement: Supplementary file 1 — Supplementary Information. [file 41598_2020_73379_MOESM1_ESM.pdf]

Supplementary Material for: Virtual zero-photon catalysis for improving continuous-variable quantum key distribution via Gaussian post-selection

Hai Zhong[1], Ying Guo[1,2,*], Yun Mao[1], Wei Ye[1,†], and Duan Huang[1]

[1]School of Computer Science and Engineering, Central South University, Changsha 410083, China
[2]State Key Laboratory of Advanced Optical Communication Systems and Networks, Shanghai Jiao Tong University, Shanghai 200240, China
[*]yingguo@csu.edu.cn
[†]yeweicsu@csu.edu.cn

ABSTRACT

This supplement contains the derivations of covariance matrixes and secret key rates for both the VZPC-based and the V1-PS-based CVQKD, respectively.

1 Derivation of Covariance matrixes for VZPC-based CVQKD

In order to obtain the analytical expression of the covariance matrix of Γ_{AB_1}, we first rewrite the state $|\Phi\rangle_{AB}$ in the form of the density operator, i.e.

$$\rho_{AB} = |\Phi\rangle_{AB}\langle\Phi|_{AB} = (1-\lambda^2)\sum_{n=0}^{\infty}\lambda^{2n}|n,n\rangle_{AB}\langle n,n|_{AB}. \tag{1}$$

Then, after the ZPC operation on mode B, the yield state $|\Phi\rangle_{AB_1}$ can be given by

$$\rho_{AB_1} = \hat{C}_0^B(1-\lambda^2)\sum_{n=0}^{\infty}\lambda^{2n}|n,n\rangle_{AB}\langle n,n|_{AB}\hat{C}_0^B = \frac{(1-\lambda^2)}{P_1}\sum_{n=0}^{\infty}(g\lambda)^{2n}|n,n\rangle_{AB_1}\langle n,n|_{AB_1}. \tag{2}$$

Using the completeness of the resulted state that $\mathrm{Tr}(\rho_{AB_1}) = 1$, we obtain $P_1 = P_Z$. Therefore, the elements of the covariance matrix Γ_{AB_1} can be calculated straightforward as follows:

$$\begin{aligned}
V_A &= \mathrm{Tr}[\rho_{AB_1}(2a^\dagger a+1)] = \frac{1-\lambda^2}{P_Z}\left[\sum_{n=0}^{\infty}(2n+1)(g\lambda)^{2n}\right] \\
&= \frac{(1-\lambda^2)(g^2\lambda^2+1)}{P_Z(1-g^2\lambda^2)} = \frac{1+VT+V-T}{1-VT+V+T}, && (3)\\
V_{B_1} &= \mathrm{Tr}[\rho_{AB_1}(2b^\dagger b+1)] = V_A = \frac{1+VT+V-T}{1-VT+V+T}, && (4)\\
C_1 &= \mathrm{Tr}[\rho_{AB_1}(ab+a^\dagger b^\dagger)] \\
&= \frac{1-\lambda^2}{P_Z}\left[\sum_{n=1}^{\infty}n(g\lambda)^{2n-1} + \sum_{m=0}^{\infty}(m+1)(g\lambda)^{2m+1}\right] \\
&= \frac{1-\lambda^2}{P_Z}\frac{2g\lambda}{[1-(g\lambda)^2]^2} = \frac{2\sqrt{T(V^2-1)}}{1-VT+V+T}. && (5)
\end{aligned}$$

With the computation results above, we can easily acquire the covariance matrixes of Γ_{AB_2} and Γ_{AB_3} for both asymptotical and finite-size frameworks. Here, we assume that the quantum channel can be characterized by the channel transmittance T_C and excess noise ξ. At the receiver, the imperfection of the detector is described by two parameters — quantum efficiency η and electrical noise ξ_{el}.

1.1 Asymptotical Case

After passing through the quantum channel, the covariance matrix of the output state ρ_{AB_2} in the asymptotical framework can be given by

$$\Gamma_{AB_2} = \begin{pmatrix} V_A\mathbf{I} & C_2\sigma_Z \\ C_2\sigma_Z & V_{B_2}\mathbf{I} \end{pmatrix}, \tag{6}$$

where $C_2 = \sqrt{T_C}C_1$, $V_{B_2} = T_C(V_{B_1} + \chi_{line})$ with $\chi_{line} = 1/T_C - 1 + \xi$. Then, the covariance matrix of the state ρ_{AB_3} is

$$\Gamma_{AB_3} = \begin{pmatrix} V_A\mathbf{I} & C_3\sigma_Z \\ C_3\sigma_Z & V_{B_3}\mathbf{I} \end{pmatrix} \tag{7}$$

with

$$V_{B_3} = \eta T_C(V_{B_1} + \chi_{line} + \chi_{det}/T_C), \tag{8}$$

$$C_3 = \sqrt{\eta}C_2, \tag{9}$$

where $\chi_{det} = (1-\eta+\xi_{el})/\eta$ for homodyne detection and $\chi_{det} = (1-\eta+2\xi_{el})/\eta$ for heterodyne detection[1]. Note, here the unit of shot noise introduced by the heterodyne detection is not absorbed into χ_{det}.

1.2 Finite-size Case

Following the derived results in Ref.[2], for the finite-size situation of our scheme, the covariance matrix of the state ρ_{AB_3} can be given by

$$\Gamma_{AB_3} = \begin{pmatrix} V_A\mathbf{I} & C_3'\sigma_Z \\ C_3'\sigma_Z & V_{B_3}'\mathbf{I} \end{pmatrix}, \tag{10}$$

where

$$V_{B_3}' = t_m^2 V_a + \sigma_m^2, C_3' = t_m C_1. \tag{11}$$

Here, t_m and σ_m are the minimally estimated value of the total transmittance and the maximally estimated value of the total noise. For homodyne detection, these two parameters can be expressed as

$$\sigma_m^2 = \sigma^2 + z_{\varepsilon_{EP}/2}\frac{\sqrt{2}\sigma^2}{\sqrt{m}}, \tag{12}$$

$$t_m = \sqrt{\eta T_C} - z_{\varepsilon_{EP}/2}\sqrt{\frac{\sigma^2}{mV_a}}, \tag{13}$$

where $\sigma^2 = \eta T_C(1+\chi_{line}+\chi_{det}/T_C)$, m is the data used for parameter estimation, $z_{\varepsilon_{EP}/2}$ is such that $[1-\mathrm{erf}(z_{\varepsilon_{EP}/2}/\sqrt{2})]/2 = \varepsilon_{EP}/2$, ε_{EP} is the failure probability of the parameter estimation and erf represents the error function defined as

$$\mathrm{erf}(x) = \frac{2}{\sqrt{\pi}}\int_0^x e^{-t^2}dt. \tag{14}$$

For heterodyne detection, t_m and σ_m^2 have the same form as the case of homodyne detection, e.g. Eqs.(13) and (12).

1.3 Composable security

Composable security proof of point-to-point one way CVQKD have been established recently[3–5]. Here, following the model built in Ref.[4], we derive the covariance matrixes needed for computing the secret key rate of our proposed scheme. Assume X and Y, which both have length of $2n$ for homodyne detection and $4n$ for heterodyne detection, the continuous variables Alice and Bob obtained before classical data post-processing. $2n$ represents the number of exchanged signals. We define robustness ε_{rob} of our CVQKD protocol the probability that the protocol aborts if the eavesdropper is inactive. Choosing the robustness $\varepsilon_{rob} \leq 0.01$, then the values of random variables $\| X \|^2$, $\| Y \|^2$, $\langle XY \rangle$ should satisfy the following inequalities[4]:

$$\| X \|^2 \leq 2n(V_A+1) + 3\sqrt{4n(V_A+1)}, \tag{15}$$

$$\| Y \|^2 \leq 2n(V_{B_3}+1) + 3\sqrt{4n(V_{B_3}+1)}, \tag{16}$$

$$\langle XY \rangle \geq 2nC_3 - 3\sqrt{n(V_A-1)(\eta T_C\chi_{tot}+1)}, \tag{17}$$

where $\chi_{tot} = 1 + \chi_{line} + \chi_{det}/T_C$, each (x_i, y_i) is modeled as identical and independent normal random variables, centered and with covariance matrix

$$\Gamma_1 = \begin{pmatrix} V_A + 1 & C_3 \\ C_3 & V_{B_3} + 1 \end{pmatrix}. \tag{18}$$

With these bounds on $\parallel X \parallel^2$, $\parallel Y \parallel^2$ and $\langle XY \rangle$, the covariance matrix needed for computing the Holevo information between Eve and Bob's measurement result can be given by[4]

$$\Gamma_m = \begin{pmatrix} \Sigma_a^{max} & \Sigma_c^{min} \\ \Sigma_c^{min} & \Sigma_b^{max} \end{pmatrix} \tag{19}$$

with

$$\Sigma_a^{max} = \frac{\parallel X \parallel^2}{2n}\left[1 + 2\sqrt{\frac{\log_2(36/\varepsilon_{PE})}{n}}\right] - 1, \tag{20}$$

$$\Sigma_b^{max} = \frac{\parallel Y \parallel^2}{2n}\left[1 + 2\sqrt{\frac{\log_2(36/\varepsilon_{PE})}{n}}\right] - 1, \tag{21}$$

$$\Sigma_c^{min} = \frac{\langle XY \rangle}{2n} - 5\sqrt{\frac{\log_2(8/\varepsilon_{PE})}{n^3}}(\parallel X \parallel^2 + \parallel Y \parallel^2), \tag{22}$$

where ε_{PE} is the maximum failure probability of parameter estimation.

2 Calculation of the secret key rate for VZPQC-based CVQKD

Given the covariance matrix derived in Section 1, we can evaluate the lower bound of the secret key rate as follows.

2.1 asymptotical security

In the case of asymptotical security, the secret key rate reads[6]

$$K = P_Z\left[\beta I_{AB} - \chi_{BE}\right], \tag{23}$$

where β is the reconciliation efficiency, I_{AB} is the mutual information shared by Alice and Bob, χ_{BE} is the maximum information available to Eve in Bob's key. For homodyne detection, the mutual information between Alice and Bob reads[6]

$$I_{AB} = \frac{1}{2}\log_2\frac{\widetilde{V}_A}{V_{A|B_3}}, \tag{24}$$

where $\widetilde{V}_A = (V_A + 1)/2$, $V_{A|B_3} = \widetilde{V}_A - C_3^2/(2V_{B_3})$. The Holevo bound χ_{BE} can be given by

$$\chi_{BE} = \sum_{i=1}^{2} G(\frac{\lambda_i - 1}{2}) - G(\frac{\lambda_3 - 1}{2}), \tag{25}$$

where $G(x) = (x+1)\log_2(x+1) - x\log_2 x$, and

$$\lambda_{1,2}^2 = \frac{1}{2}\left[\Delta \pm \sqrt{\Delta^2 - 4D^2}\right], \tag{26}$$

$$\lambda_3^2 = V_A(V_A - C_3^2/V_{B_3}), \tag{27}$$

with the notions

$$\Delta = V_A^2 + V_{B_3}^2 - 2C_3^2, \tag{28}$$

$$D = V_A V_{B_3} - C_3^2. \tag{29}$$

For the case of heterodyne detection, the mutual information shared by Alice and Bob reads[6]

$$I_{AB} = \log_2\frac{\widetilde{V}_A}{\widetilde{V}_{A|B_3}}, \tag{30}$$

where $\widetilde{V}_{A|B_3} = \widetilde{V}_A - C_3^2/(4\widetilde{V}_{B_3})$ with $\widetilde{V}_{B_3} = (V_{B_3} + 1)/2$. The calculation process of the Holevo bound χ_{BE} is similar to the homodyne counterpart. The acquirement of eigenvalues of λ_1 and λ_2 is similar to Eq. (27). The eigenvalue of λ_3 is given as[6]

$$\lambda_3 = V_A - \frac{C_3^2}{V_{B_3} + 1}. \tag{31}$$

2.2 finite-size case

In the finite-size scenario, the secret key rate reads[2]

$$K = P_Z \cdot \frac{n}{N}\left[\beta I_{AB} - \chi_{BE} - \Delta(n)\right], \tag{32}$$

where N is the total exchanged data, n is the data used for generating row key ($m = N - n$), $\Delta(n)$ is a parameter related with privacy amplification and is given by

$$\Delta(n) = 7\sqrt{\frac{\log_2(2/\varepsilon_{sm})}{n}} + \frac{2}{n}\log_2(1/\varepsilon_{PA}), \tag{33}$$

where ε_{sm} is a *smoothing* parameter, ε_{PA} is the failure probability of the privacy amplification procedure. The calculations of I_{AB} and χ_{BE} are similar to the asymptotical case, just replace C_3 and V_{B_3} to C_3' and V_{B_3}'.

2.3 Composable security

In the composable security framework, the secret key rate can be given as[4]

$$K = P_Z(1-\varepsilon_{rob})K' \tag{34}$$

with

$$K' = \beta I_{AB} - f(\Sigma_a^{max}, \Sigma_b^{max}, \Sigma_c^{min}) - \frac{1}{2n}\left(\Delta_{AEP} + \Delta_{ent} + 2\log_2\frac{1}{2\bar{\varepsilon}}\right), \tag{35}$$

where $f(\Sigma_a^{max}, \Sigma_b^{max}, \Sigma_c^{min})$ is the Holevo information between Eve and Bob, $\bar{\varepsilon}$ is the security parameter comes fromthe leftover hash lemma. Δ_{AEP} is given by

$$\Delta_{AEP} = \sqrt{2n}(d+1)^2 + 4(d+1)\sqrt{2n}\log_2(\frac{2}{\varepsilon_{sm}^2}) + 2\sqrt{2n}\log_2(\frac{2}{\varepsilon^2\varepsilon_{sm}}) + 4\frac{\varepsilon_{sm}d}{\varepsilon}, \tag{36}$$

where ε_{sm} the smoothing parameter entering the smooth conditional min-entropy, d the number of bits on which each measurement result is encoded, ε is the security parameter of the QKD protocol given by

$$\varepsilon = \sqrt{\varepsilon_{PE} + \varepsilon_{cor} + \varepsilon_{ent}} + 2\varepsilon_{sm} + \bar{\varepsilon}. \tag{37}$$

For homodyne detection, the mutual information between Alice and Bob reads

$$I_{AB} = \frac{1}{2}\log_2\left(1 + \frac{\eta T_C V_a}{\eta T_C \chi_{tot}}\right) = \frac{1}{2}\log_2\left(1 + \frac{V_a}{\chi_{tot}}\right). \tag{38}$$

The Holevo information between Eve and Bob is given by

$$f(\Sigma_a^{max}, \Sigma_b^{max}, \Sigma_c^{min}) = \sum_{i=1}^{2} G(\frac{\nu_i - 1}{2}) - G(\frac{\nu_3 - 1}{2}), \tag{39}$$

where

$$\nu_{1,2}^2 = \frac{1}{2}\left[\Delta_1 \pm \sqrt{\Delta_1^2 - 4D_1^2}\right], \tag{40}$$

$$\nu_3^2 = \Sigma_a^{max}\left(\Sigma_a^{max} - \frac{(\Sigma_c^{min})^2}{\Sigma_b^{max}}\right) \tag{41}$$

with the notion

$$\Delta_1 = (\Sigma_a^{max})^2 + (\Sigma_b^{max})^2 - 2(\Sigma_c^{min})^2, \tag{42}$$

$$D_1 = \Sigma_a^{max}\Sigma_b^{max} - (\Sigma_c^{min})^2. \tag{43}$$

As the length of raw key between Alice and Bob is $2n$ when performing homodyne detection, the quantity Δ_{ent} should be expressed as

$$\Delta_{ent} = \log_2\frac{1}{\varepsilon} + \sqrt{4n\log_2^2(2n)\log_2\frac{2}{\varepsilon_{sm}}}. \tag{44}$$

For heterodyne detection, the mutual information between Alice and Bob reads

$$I_{AB} = \log_2\left(1+\frac{\eta T_C V_a}{1+\eta T_C \chi_{tot}}\right). \tag{45}$$

The Holevo information between Eve and Bob has the same form with Eq. (39), while the eigenvalue ν_3 should be replaced as

$$\nu_3 \quad = \quad \Sigma_a^{max} - \frac{(\Sigma_c^{min})^2}{\Sigma_b^{max}+1}. \tag{46}$$

As the length of raw key between Alice and Bob is $4n$ when performing heterodyne detection, the quantity Δ_{ent} should be expressed as

$$\Delta_{ent} = \log_2\frac{1}{\varepsilon} + \sqrt{8n\log_2^2(4n)\log_2\frac{2}{\varepsilon_{sm}}}. \tag{47}$$

3 Calculations of covariance matrixes and the secret key rate for CVQKD with virtual 1-photon subtraction

For comparison with our proposed scheme, here, we give the calculations of covariance matrixes and the secret key rate for CVQKD with virtual 1-photon subtraction. After 1-photon subtraction operation, the covariance matrix of the state ρ_{AB_1} is[7]

$$\Gamma_{AB_1}^{PS} = \begin{pmatrix} V_A^{PS}\mathbf{I} & C_1^{PS}\sigma_Z \\ C_1^{PS}\sigma_Z & V_{B_1}^{PS}\mathbf{I} \end{pmatrix} \tag{48}$$

with

$$V_A^{PS} \quad = \quad \frac{3(V+1)+TV-T}{V+1-TV+T}, \tag{49}$$

$$V_{B_1}^{PS} \quad = \quad V_b^{PS}+1 = \frac{V+1+3T(V-1)}{V+1-TV+T}, \tag{50}$$

$$C_1^{PS} \quad = \quad \frac{4\sqrt{T(V^2-1)}}{V+1-TV+T}, \tag{51}$$

$$V_b^{PS} \quad = \quad \frac{4T(V-1)}{V+1-TV+T}. \tag{52}$$

The covariance matrix of the state ρ_{AB_3} and the deviations of secret key rates for all the two mentioned security frameworks are similar to the VZPC-based protocol, just replace V_A, V_{B_1}, C_1 and V_a to V_A^{PS}, $V_{B_1}^{PS}$, C_1^{PS} and V_b^{PS}.

Note, the 1-photon subtraction (1-PS) operation is a non-Gaussian operation. The non-Gaussian behavior of 1-PS makes the conventional calculation methods of secret key rate of Gaussian modulated coherent state CVQKD protocol inapplicable. Usually, one still assume that the eavesdropper performs collective Gaussian attack (optimal attack for Gaussian channel but may not the optimal one for non-Gaussian channel) for such non-Gaussian protocols. Under this assumption, the calculation of secret key rate for 1-PS in references is based on the Gaussian approximation. According to the extemality of Gaussian quantum states[8], the secret key rate of the 1-PS state (non-Gaussian state) is no less than a Gaussian state which has the same covariance matrix. Ideally, one can calculate the exact mutual information between Alice and Bob and the exact Holevo information between Bob and Eve (reverse reconciliation) by directly using the conditional distribution of the 1-PS state. But it is extremely cumbersome. Therefore, the numerical results shown in the main text for 1-PS are based on the above Gaussian hypothesis.

References

1. Fossier, S., Diamanti, E., Debuisschert, T., Tualle-Brouri, R. & Grangier, P. Improvement of continuous-variable quantum key distribution systems by using optical preamplifiers. *J. Phys. B: At., Mol. Opt. Phys.* **42**, 114014, DOI: 10.1088/0953-4075/42/11/114014 (2009).

2. Leverrier, A., Grosshans, F. & Grangier, P. Finite-size analysis of a continuous-variable quantum key distribution. *Phys. Rev. A* **81**, 062343, DOI: 10.1103/PhysRevA.81.062343 (2010).

3. Furrer, F. *et al.* Continuous variable quantum key distribution: Finite-key analysis of composable security against coherent attacks. *Phys. Rev. Lett.* **109**, 100502, DOI: 10.1103/PhysRevLett.109.100502 (2012).

4. Leverrier, A. Composable security proof for continuous-variable quantum key distribution with coherent states. *Phys. Rev. Lett.* **114**, 070501, DOI: 10.1103/PhysRevLett.114.070501 (2015).

5. Leverrier, A. Security of continuous-variable quantum key distribution via a gaussian de finetti reduction. *Phys. Rev. Lett.* **118**, 200501, DOI: 10.1103/PhysRevLett.118.200501 (2017).

6. Sánchez, G.-P. & Raúl. Quantum information with optical continuous variables: from bell tests to key distribution (2007).

7. Li, Z. *et al.* Non-gaussian postselection and virtual photon subtraction in continuous-variable quantum key distribution. *Phys. Rev. A* **93**, 012310, DOI: 10.1103/PhysRevA.93.012310 (2016).

8. Navascues, M., Grosshans, F. & Acin, A. Optimality of gaussian attacks in continuous-variable quantum cryptography. *Phys. Rev. Lett.* **97**, 190502 (2006).
